# GAGA for nonreciprocal emitters: genetic algorithm gradient ascent optimization of compact magnetophotonic crystals

**DOI:** 10.1515/nanoph-2023-0598

**Published:** 2024-01-08

**Authors:** Hannah Gold, Simo Pajovic, Abhishek Mukherjee, Svetlana V. Boriskina

**Affiliations:** Massachusetts Institute of Technology, Cambridge, USA

**Keywords:** Weyl semimetals, nonreciprocity, genetic algorithm, mid-infrared, subwavelength, energy and sustainability

## Abstract

Fundamental limits of thermal radiation are imposed by Kirchhoff’s law, which assumes the electromagnetic reciprocity of a material or material system. Thus, breaking reciprocity can enable breaking barriers in thermal efficiency engineering. In this work, we present a subwavelength, 1D photonic crystal composed of Weyl semimetal and dielectric layers, whose structure was optimized to maximize the nonreciprocity of infrared radiation absorptance in a planar and compact design. To engineer an ultra-compact absorber structure that does not require gratings or prisms to couple light, we used a genetic algorithm (GA) to maximize nonreciprocity in the design globally, followed by the application of the numerical gradient ascent (GAGA) algorithm as a local optimization to further enhance the design. We chose Weyl semimetals as active layers in our design as they possess strong, intrinsic nonreciprocity, and do not require an external magnetic field. The resulting GAGA-generated 1D magnetophotonic crystal offers high nonreciprocity (quantified by absorptance contrast) while maintaining an ultra-compact design with much fewer layers than prior work. We account for both s- and p-polarized absorptance spectra to create a final, eight-layer design suitable for thermal applications, which simultaneously minimizes the parasitic, reciprocal absorptance of s-polarized light.

## 1 Introduction

In radiative heat transfer, Kirchhoff’s law of radiation [1] states that the spectral directional emissivity of a surface equals its spectral directional absorptivity: $\epsilon \left(\omega ,\hat{s}\right)=\alpha \left(\omega ,\hat{s}\right)$ , where *ω* is the angular frequency and $\hat{s}$ is the directional unit vector. This equality is often thought of as being a consequence of the second law of thermodynamics, but in fact, it is a consequence of electromagnetic reciprocity. It has indeed been shown theoretically and experimentally that systems that break electromagnetic reciprocity (i.e., nonreciprocal systems) violate Kirchhoff’s law of radiation, $\epsilon \left(\omega ,\hat{s}\right)\neq \alpha \left(\omega ,\hat{s}\right)$ [2], [3]. For example, time reversal symmetry-breaking materials such as magnetic materials are nonreciprocal [4]. Kirchhoff’s law implies an inherent loss of energy because any absorber must emit back through the same $\left(\omega ,\hat{s}\right)$ channel. Therefore, in theory, one should be able to improve the efficiency of thermally-emitting devices by breaking this symmetry. Nonreciprocity and the breakdown of Kirchhoff’s law are significant because they dissolve assumptions that impose theoretical limits on radiative and absorptive energy conversion efficiency and can improve efficiency for devices such as thermal emitters [5], [6], [7], solar photovoltaic cells [8], [9], and antennas [10]–[14]. Nonreciprocity also allows for the engineering of optical isolators [15].

In recent years, the most popular route toward engineering nonreciprocity in thermal emitters – which operate in the mid- to far-infrared (IR) spectrum at room temperature – has been using magnetic materials. A recently discovered class of materials called time reversal symmetry-breaking Weyl semimetals (WSMs) [16] has received considerable attention because they are predicted to exhibit strong nonreciprocity in the mid-IR without the need for an external magnetic field, a requirement that can be cumbersome and impractical. In these materials, the flux of Berry curvature between Weyl nodes of opposite chirality acts like a pseudo-magnetic field in momentum space, which gives rise to the anomalous Hall effect and thereby large off-diagonal components of the dielectric tensor (comparable in magnitude to the diagonal components) [17]. This results in strong nonreciprocity, which we leverage in our design of a WSM-based 1D magnetophotonic crystal.

Prior to the discovery of WSMs, popular material platforms for engineering nonreciprocal devices included narrow bandgap semiconductors such as InAs and InSb under external magnetic fields, and magnetic materials such as YIG [18]–[26]. In these systems, nonreciprocal versions of surface waves, e.g., surface plasmon polaritons and Tamm plasmons, are leveraged to achieve nonreciprocal optical properties. To facilitate coupling of propagating waves to the surface modes, multilayered absorbers have been engineered [22], [23], [24], along with the use of the conventional coupling structures such as prisms and gratings [2], [18], [19], [20], [21]. The use of coupling prisms prevents realization of chip-integrated absorber or emitter designs and may limit the range of operational wavelengths by the availability of transparent prism materials. Fabrication of gratings calls for e-beam or optical lithography techniques to be used, increasing the absorber cost and complexity, and potentially limiting its footprint.

Likewise, the inherently strong nonreciprocity of WSM surface modes [7], [27] can be further enhanced by engineering multilayer structures or gratings to facilitate optical energy coupling to and from these high-momentum modes. However, most designs of WSM-based nonreciprocal absorbers or emitters proposed to date have complicated geometries and either requires grating couplers or dozens of planar layers to achieve functionality. For example, an absorber structure presented in Ref. [28] enhances nonreciprocity via a 49-layer multilayer design consisting of a WSM with periodic dielectric 1D photonic crystals on either side. In Ref. [29], a grating structure and mirror combination is used on either side of a WSM, while [30] presents a 42-layer structure combining the local field enhancement of Tamm plasmon states with a WSM.

Table 1 and Figure 1a summarize the results of recent studies of nonreciprocal absorbers/emitters utilizing either magneto-optic materials or WSMs as active layers. The data in Table 1 show that nonreciprocal structures utilizing magneto-optic materials typically require external magnetic fields of 0.3–10 T to achieve nonreciprocal absorptance. The table also introduces a typical figure of merit (FOM) – the absorptance contrast – used to quantify the level of nonreciprocity in the emitter design. The absorptance contrast is typically defined as:

(1) Δα=max(|α(ω,θ)−α(ω,−θ)|),

and in previous designs ranges from about 45 %–95 %, depending on the complexity of the structure.

**Table 1:** Comparison of nonreciprocal thermal emitter or absorber designs reported in the literature. For references reporting multiple designs with varying parameters, the set of parameters yielding the largest absorptance contrast was used. WebPlotDigitizer [31] was used to extract the absorptivity contrast from graphical data when it was not explicitly provided.

	[18]	[19]	[29]	[32]	[7]	[20]	[33]	[22]	[34]	[30]	[35]	[36]	[28]	[37]	[38]	Our Work
$\begin{array}{c} \text{Peak}\\ \text{wavelength}\\ (\mu \text{m)} \end{array}$	∼16	∼16	∼15	∼10	∼20	∼25	∼10	∼15	∼16	∼7	∼10	∼15	∼10	∼5	∼9	∼12
$\begin{array}{c} \text{Angle of Incidence}\\ \left(\pm \;{}^{\circ}\right) \end{array}$	61.28	54.75	30	30	60	65	60	56	50	18	32	62	58	15	45	55
$\begin{array}{c} \text{External}\\ \text{magnetic}\\ \text{field}\left(T\right) \end{array}$	3	2	0	10	0	0.3	3	3	3	0	0	0	0	0	0	0
$\begin{array}{c} \text{Active}\\ \text{material}(\text{s}) \end{array}$	n-InAs	InAs	WSM	$\begin{array}{c} \text{Magneto}-\text{optical}\\ model \end{array}$	WSM	InAs	InAs, prism	InAs	InSb	ITO, WSM	$\begin{array}{c} \text{WSM, defect}\\ \text{layer} \end{array}$	WSM	WSM	WSM	WSM	WSM
$\begin{array}{c} \text{Lossy}\\ \text{dielectric?} \end{array}$	$\begin{array}{c} \text{No dielectric,}\\ \text{used PEC} \end{array}$	Yes	No	Yes	Yes	Yes	No	No	Yes	No	No	No	No	No	Yes	Yes
$\begin{array}{c} \text{Coupling}\\ \text{device?} \end{array}$	$\begin{array}{c} \text{Yes, n}-\text{InAs}\\ grating \end{array}$	$\begin{array}{c} \text{Yes, Si}\\ grating \end{array}$	$\begin{array}{c} \text{Yes, Si}\\ grating \end{array}$	$\begin{array}{c} \text{Yes, SiC}\\ grating \end{array}$	$\begin{array}{c} \text{Yes, low loss}\\ \text{dielectric grating} \end{array}$	$\begin{array}{c} \text{Yes, SiC}\\ grating \end{array}$	Yes, prism	No	No	No	No	No	No	No	No	No
$\begin{array}{c} \text{Planar}\\ \text{multilayer?}(\text{\#}\\ \text{of layers}) \end{array}$	No	No	No	No	No	No	No	17	6	42	23	49	22	100	3	8
$\begin{array}{c} \text{Absorptance}\\ \text{contrast}\left(\Delta \alpha \right) \end{array}$	93.17 %	91.0 %	93.8 %	44.8 %	56.3 %	91.0 %	86.0 %	92.0 %	85.5 %	95.7 %	93.6 %	92.3 %	95.5 %	98.3 %	53.0 %	95.3 %

**Figure 1:**
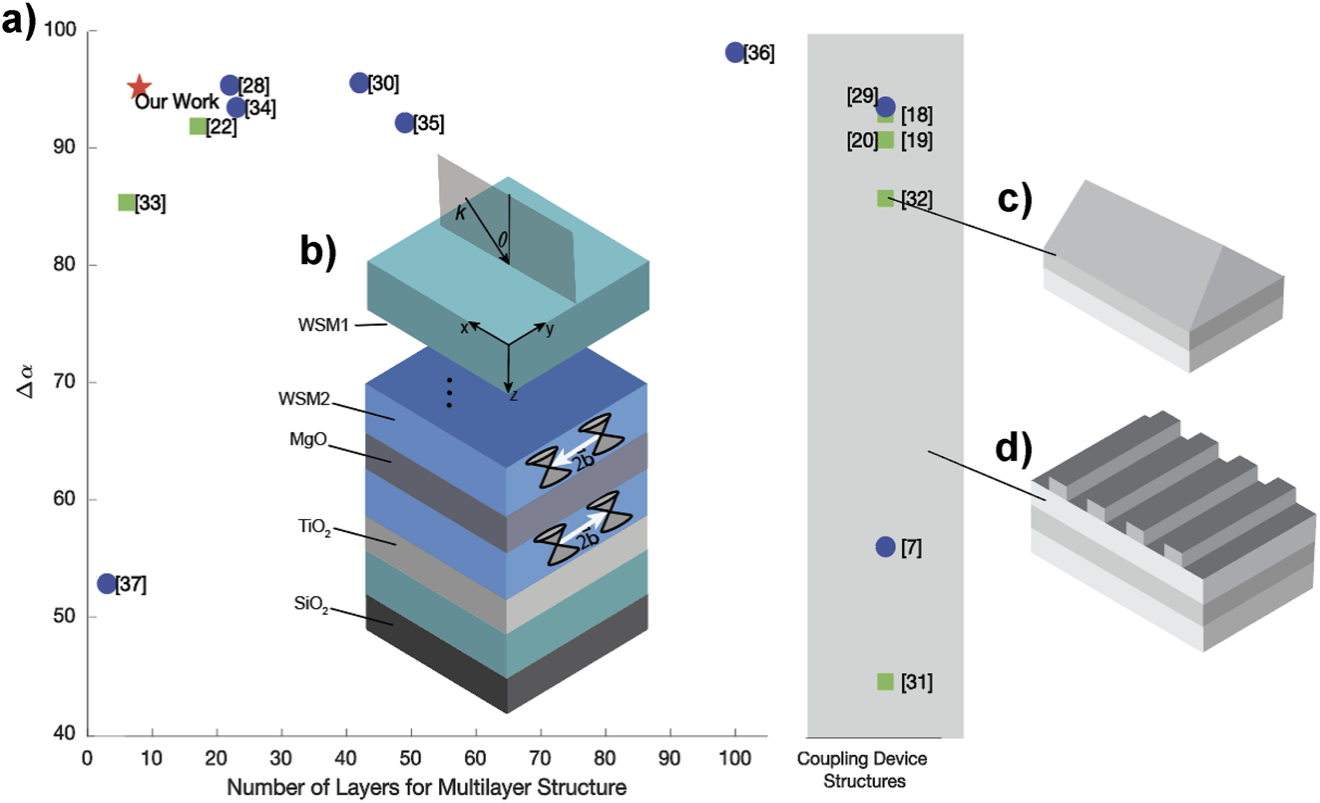
Design configuration and comparison withprevious work. (a) Comparison of the level of nonreciprocity (quantified as the p-polarized absorptivity contrast Δ*α*, see Eq. (1)) of the designs listed in Table 1, as a function of the design complexity (quantified as the number of layers and/or additional coupling elements). The data for the designs with couplers and those without (the multilayer structures) are grouped separately. The green squares label the absorbers incorporating conventional magnetooptical materials that require a magnetic field bias; the blue circles correspond to the designs based on Weyl semimetals; and the red star shows the result of this work. (b) (Inset) Generalized design configuration that includes the materials in our multilayer structure, Weyl semimetal 1 (WSM1), Weyl semimetal 2 (WSM2), MgO, SiO_2_, and TiO_2_. The wavevector, $\vec{k}$ (pointing in the *xz*-plane) of the incident wave forms polar angle *θ* with the absorber surface normal, and the white arrow with $2\vec{b}$ (pointing in the ±*y*-direction) is the Weyl node separation vector. (c) and (d) Schematics of a grating-based and prism-based structure, respectively; (c) indicates the only prism design and (d) shows that the other coupling structures are grating-based.

In this work, we develop a genetic algorithm coupled with the gradient ascent technique (GAGA) to optimize the nonreciprocal absorptivity and violation of Kirchhoff’s law in a 1D planar magnetophotonic crystal composed of dielectric and magnetic WSM thin films. The optimized design achieves a large nonreciprocal absorptivity contrast in the near-infrared at opposing polar angles of incidence, ±55°. Our optimization process leverages a unique figure of merit that accounts for both s- and p-polarized light, which is vital for applications to thermal radiation since it consists of a random mixture of both polarizations. The final absorber design enables strong nonreciprocity without the need for an external magnetic field, and accounts for manufacturability by limiting itself to thicknesses that can be realistically fabricated and using fewer layers than prior designs with comparable FOMs.

## 2 Design specifications

Our work considers a 1D magnetophotonic crystal consisting of dielectric and magnetic materials, as illustrated in Figure 1b. Light with wavevector $\vec{k}$ is incident in the *xz*-plane at a polar angle *θ* onto the *N*-layer absorber structure. This 1D magnetophotonic crystal is constructed from a library of materials for the optimization algorithm to choose from; for instance, the teal and blue colors correspond to two different WSM materials included in our design: WSM1 and WSM2, to be distinguished later in this section. The differing shades of gray are used for the dielectrics SiO_2_, TiO_2_, and MgO. Our goal is to optimize the magnetophotonic crystal design to maximize nonreciprocity at a given wavelength, i.e., to achieve a high contrast in absorptance between two opposing angles, chosen as −55° and 55° in this study.

The dielectric materials included in the system design exhibit various levels of loss in the mid- to far-IR spectral range, and to account for this loss we used wavelength-dependent complex dielectric functions in our calculations. Some references in Table 1 have neglected losses in dielectric constituents in the same or similar spectral range. Because of the phonon-driven absorptance in the IR spectral range and a possibility of surface phonon–polariton modes forming on material interfaces, these losses cannot be ignored, and are included in our model. The complex permittivities of SiO_2_, TiO_2_, MgO are given in references [39]–[41], respectively, and shown in the Appendix B Figure 1.

The simplest Weyl semimetals featuring two Weyl nodes (i.e., discrete points in the momentum space where their conduction and valence bands touch) have been chosen as magnetic materials (WSM1 and WSM2) in our design. Figure 1b shows the coordinate system and the direction of the Weyl node separation vectors 2 $\vec{b}$ in representative WSM layers. In reflective nonreciprocal systems based on magnetic multilayer structures, the magnetization must have an in-plane component. In this situation, the system can be examined in two limiting configurations: the Voigt configuration, in which light propagates perpendicular to the Weyl node separation vector 2 $\vec{b}$ ( $\vec{k}⊥\;2\vec{b}$ , where $\vec{k}$ is the wavevector), and the Faraday configuration, in which light propagates parallel to the Weyl node separation $\left(\vec{k}\|2\vec{b}\right)$ . As a result of symmetry, the Faraday configuration is reciprocal, and the Voigt configuration maximizes nonreciprocity, so our system is designed around the Voigt configuration. In this configuration, the eigenmodes supported by the magnetic materials are classified as purely s- or p-polarized, and only p-polarized ones exhibit nonreciprocal behavior. However, as the s-polarized modes can be thermally populated, they contribute to thermal radiation. Their reciprocal contribution may limit the nonreciprocal response of the thermal absorber or emitter driven by p-polarized light. Thus, we included s-polarized waves in our FOM definition, to be discussed in Section 3.1.

In the Voigt configuration, the Weyl nodes are oriented either parallel or antiparallel to the *y*-direction. This means the dielectric tensor takes the following form:

(2) $${\bar{\bar{\varepsilon }}}_{\text{Weyl}}\left(\omega \right)=\left[\begin{array}{ccc} \varepsilon_{xx} & 0 & i\varepsilon_{a}\\ 0 & \varepsilon_{yy} & 0\\ -i\varepsilon_{a} & 0 & \varepsilon_{zz} \end{array}\right].$$

In Eq. (2), $\varepsilon_{a}=be^{2}/\left(2\pi^{2}\hbar \varepsilon_{0}\omega \right)$ , where *ɛ*_0_ is the permittivity of free space, and *b* refers to the Weyl node separation, $2b=2|\vec{b}|$ . The off-diagonal component, *ɛ*_*a*_, is a consequence of the anomalous Hall effect (AHE). The diagonal terms *ɛ*_*ii*_ (with *i* = *x*, *y*, *z*) are calculated by using the Kubo–Greenwood formalism within the random phase approximation to a two-band model with spin degeneracy [42]:

(3) $$\begin{array}{ll} \sigma_{ii} & =\frac{r_{s}g}{6}\Omega G\left(\Omega /2\right)+\frac{r_{s}g}{6\pi \omega }\left\{\frac{4}{\Omega }\left[1+\frac{\pi^{2}}{3}{\left(\frac{k_{B}T}{E_{F}\left(T\right)}\right)}^{2}\right]\right.\\ & \;\left.+8\Omega \overset{\xi_{c}}{\underset{0}{\int }}\frac{G\left(\xi \right)-G\left(\Omega /2\right)}{\Omega^{2}-4\xi^{2}}\xi \text{d}\xi \right\}, \end{array}$$

The directional conductivity, *σ*_*ii*_ is substituted into $\varepsilon_{ii}=\varepsilon_{ii}\left(\omega \right)=\varepsilon_{b}+i\frac{\sigma_{ii}\left(\omega \right)}{\frac{\hbar \omega }{E_{F}}}$ , where *ɛ*_*b*_ is the background dielectric constant. Note that the reason why *σ*_*ii*_ is divided by a dimensionless frequency is because Eq. (3) assumes *σ*_*ii*_ is dimensionless. In Eq. (3), *E*_*F*_ is the Fermi energy, $\Omega =\hbar \left(\omega +i\tau^{-1}\right)/E_{F}$ is the normalized complex frequency, *τ* ^−1^ is the Drude damping rate, *G*(*E*) = *n*(−*E*) − *n*(*E*) (where *n*(*E*) is the Fermi distribution), *r*_*s*_ = *e* ^2^/4*πϵ*_0_ *ℏv*_*F*_ is the effective fine-structure constant, *v*_*F*_ is the Fermi velocity, *g* is the number of Weyl points, and *ξ*_*c*_ = *E*_*c*_/*E*_*F*_, where *E*_*c*_ is the energy cutoff signifying the point where the band dispersion is no longer linear [42].

Guided by references [5], [43], [44], we use the following parameters to model WSM optical properties: *ɛ*_*b*_ = 6.2, *ξ*_*c*_ = 3, *τ* = 1000 fs, *g* = 2, *b* = 8.5 × 10^8^ m^−1^, and *E*_*F*_ = 0.15 eV at *T* = 300 K. Two values of the Fermi velocity are used to create two distinct WSM types, which are referenced later as WSM1 and WSM2. WSM1 has *v*_*F*_ = 1.3 × 10^5^ m/s and serves as a model for Co_3_Sn_2_S_2_ [45], [46], and WSM2 has *v*_*F*_ = 1.2 × 10^4^ m/s, and serves as a model for Co_2_MnGa [47]. The diagonal and off-diagonal components of the WSM dielectric tensors are plotted in the Appendix B Figure 2a and b.

Along with the material type, there are several additional parameters which are variable in this work: the thickness of each layer in the structure, the material pairing, and the direction of the Weyl node separation ( $+\hat{y}$ or $-\hat{y}$ ) (to be discussed in Section 2.1). To generate an initial structure design to be further optimized, the chosen materials were paired as several potential material pairings: SiO_2_, TiO_2_, and MgO, each with either WSM. Each pair was randomly selected with equal probability from the first layer to the last until 5 pairs (or 10 layers) were chosen. These dielectrics serve as spacer materials. Dielectric materials are usually placed above and below the WSM or magnetooptical material so that multiple reflections can occur at the top and bottom interfaces of the dielectric layer, forming guided modes in the structure. The higher optical density of states provided by the guided modes allows for resonant amplification of the nonreciprocal surface modes on the WSM-dielectric interface.

Magnetic WSMs show a strong nonreciprocal response in the mid-infrared regime [6] as SPP modes are expected to exist at dielectric-WSM interfaces at these frequencies. The wavelength range used in this study spans the mid- to far-infrared (from 10 μm to 22.5 μm). The planar layers thickness values are randomly selected from 400 linearly-spaced points from 60 nm to 450 nm. These thicknesses are rounded to the nearest nanometer when creating the final design. It is important to note that the minimum thickness of the individual WSM layers included in our design allows for the WSMs to maintain their magnetic properties. In experimentally fabricated thin-films of Co_2_MnGa, large anomalous Hall conductivity was measured in films with thicknesses from 10 nm to 80 nm [48], [49]. The ferromagnetism of Co_3_Sn_2_S_2_ was shown to be robust in ultra-thin films [50]. A minimum design thickness of 60 nm chosen in this work ensures that the WSM materials are likely to exhibit anomalous Hall conductivity. The chosen range of allowed thicknesses is also based on practical fabricability; methods such as chemical vapor deposition [51], [52], [53], electron-beam evaporation [54]–[57], and sputtering [58], [59], [60] are all acceptable and common methods used to fabricate these materials with such a thickness range. As an example, references [61], [62] support the fabricability of our work by showing how MgO can be grown on or under Co_2_MnGa.

By choosing a multilayer structure design, we aim to avoid using gratings and prisms, which allow to overcome the momentum mismatch between propagating photons and SPPs, and to couple light into or out of the SPP modes supported by a magnetooptical material interface (see Figure 1c and d). These coupling devices have been used in the design of some of nonreciprocal emitters as shown by several references listed in Table 1. However, typical manufacturing processes utilized to create these geometries are more time- and cost- intensive than the planar multilayer deposition techniques. Standard lithography can be used to create structures similar to the one shown in Figure 1c, which requires multiple intermediate steps for creating a mask, curing and baking photoresist, etching, etc. The prisms, although not particularly difficult to fabricate [63], are quite bulky and impractical for on-chip integrated thin devices.

### 2.1 Symmetry as a design consideration

Our optimization process uses the Weyl node separation direction, $2\vec{b}$ , as an important degree of freedom in the search for the optimum nonreciprocal emitter design. The intuition behind this approach is based on the fact that configurational symmetry-breaking – that is, breaking of the macroscopic symmetry of the system as a whole, as opposed to microscopic material symmetries – plays a major role in maximizing the nonreciprocal behavior of any photonic system. Generally speaking, a system is reciprocal under the conditions of (1) linear constitutive relations (e.g., $\vec{D}=\varepsilon \vec{E}$ ) as well as (2) symmetric and (3) time-invariant property tensors [4]. Materials with broken inversion symmetry can lift the first requirement, while materials with broken time-reversal symmetry can lift the other two. Notably, magnetic materials can have antisymmetric dielectric tensors, breaking condition (2). The asymmetry of the dielectric tensor of WSMs is one manifestation of breaking reciprocity conditions (2) due to breaking time-reversal symmetry [6], [7].

While a material that lifts one or more of the three conditions of reciprocity can be said to be nonreciprocal, the effects of nonreciprocity will only manifest if the system possesses broken configurational symmetry as well. An example of this is bulk plasmons in WSMs having a reciprocal dispersion relation $\omega \left(\vec{k}\right)=\omega \left(\vec{-\vec{k}}\right)$ , while surface plasmons have a nonreciprocal dispersion relation $\omega \left(\vec{k}\right)\neq \omega \left(\vec{-\vec{k}}\right)$ because the surface breaks the symmetry of the bulk [64], [65].

Proper choice of the direction of the Weyl node separation vector $2\vec{b}$ in each WSM layer within the absorber allows breaking the configurational symmetry of the magnetophotonic crystal as a whole. Explicitly, when the node separation vector of each WSM layer flips direction, the signs of both *iɛ*_*a*_ in Eq. (2) are simultaneously being changed and, depending on each WSM layer placement within the magnetophotonic crystal, configurational symmetry can be broken and nonreciprocity maximized. The pseudo-vectorial nature of 2 $\vec{b}$ , acting as an internal magnetization in place of an external magnetic field, is what allows configurational symmetry to be broken upon coordinate inversion.

To demonstrate how configurational symmetry affects nonreciprocity in a multilayer system, we start with an example of two semi-infinite WSM slabs with material parameters corresponding to those of WSM1 and parallel node separation directions, which are separated by a 1 μm airgap (see Figure 2a). Although the SPP modes on each interface separately are nonreciprocal, this structure supports reciprocal coupled surface modes. The reason for this is that in the case of two semimetal-dielectric interfaces separated by a nanoscale distance, the surface waves supported by each interface can couple to each other. The reciprocity or lack thereof of the hybrid modes formed as a result of this coupling depends on the configurational inversion symmetry of the two-interface geometry [65], [66] The nonreciprocal SPP mode on each interface cannot couple to the identical nonreciprocal mode on the other interface if their field rotation directions do not match [64], [67]. However, if the system configurational symmetry is broken due to anti-parallel internal nodal directions of the two WSMs, nonreciprocity is achieved since the surface modes can couple (see Figure 2b). We illustrate the fundamental difference between these two configurations by comparing their SPP dispersion curves in Figure 2a and b. The dispersion equations for this structure were derived in Ref. [68] (Figure 3).

**Figure 2:**
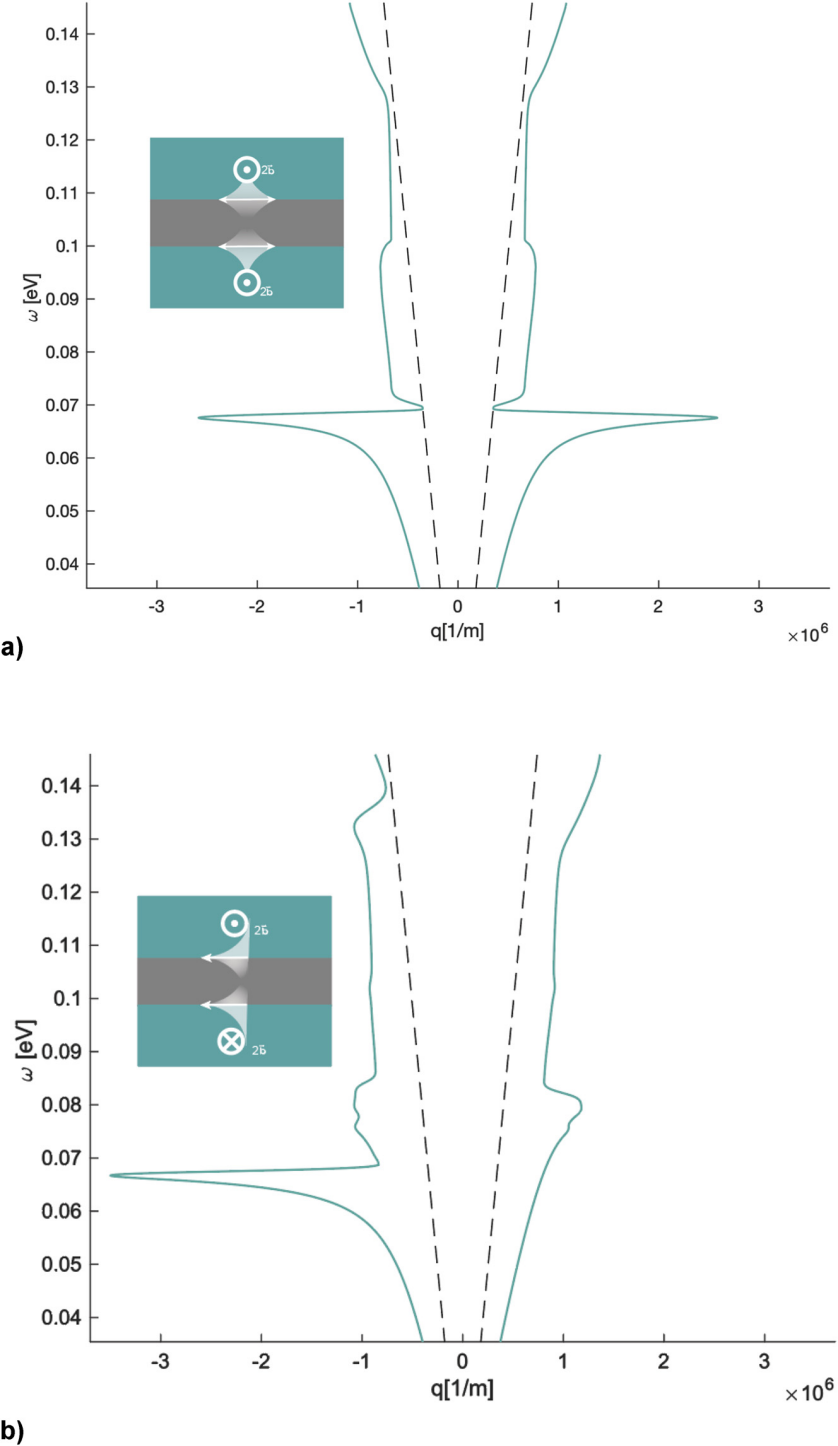
Coupled SPP dispersion curves with and without configurational symmetry as a function of in-plane wavevector. Insets: slot-waveguide structures in the Voigt configuration. (a) Two WSM slabs (with WSM1’s parameters) with the same nodal separation direction separated by a 1 μm airgap. The dispersion relation is symmetric about *k*_*y*_ = 0, meaning the coupled SPPs are reciprocal. (b) Same as (a) except the node separation vectors are pointing in the opposite directions. The dispersion relation is asymmetric about *k*_*y*_ = 0, meaning the coupled SPPs are nonreciprocal as a result of broken configurational symmetry. The dashed lines in both plots are the light line.

**Figure 3:**
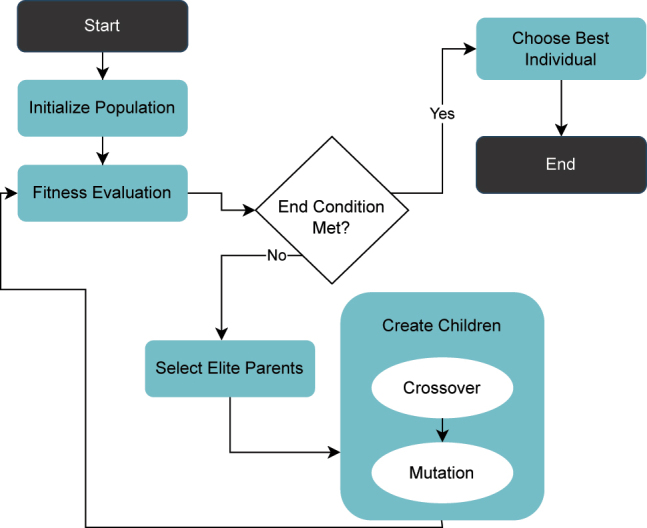
Flowchart of the genetic algorithm process. First the GA starts with initializing a population. The individuals in the population are each evaluated for their “fitness” and subsequently the best of which are selected for (assuming the end condition is not met). After this phase, children are created from the best parents through crossover and mutation before becoming the new population to be evaluated.

A similar degree of freedom – analogous to the direction of internal magnetization of Weyl semimetal defined by the $2\vec{b}$ vector – can in theory be explored in other magnetooptical materials, by changing the direction of the external magnetic field at each layer. However, it is extremely challenging to apply an external magnetic field that is highly confined and alternates in the space of a few nanometers of thickness.

The ability to use the mutual orientation of the Weyl node separation vectors to tune the optical properties of multilayer structures has been already recognized and exploited in other photonic system designs. For example [43] shows that a twisting angle between two parallel WSM slabs can be changed to control the near-field radiative heat transfer between them. Additionally, [69] used the alternating magnetic directions of two bismuth iron garnet layers with opposite magnetization and one SiO_2_ to produce one way total reflection, and references [44], [70] used Weyl semimetals in a multilayer structure with alternating Weyl node separation directions to achieve more compact optical isolator designs. Detailed discussions of the symmetry of nonreciprocal 1D magnetophotonic crystals in the context of a group theory argument can be found in Ref. [71] and theoretical nonreciprocity constraints are placed on systems such the many body radiative systems seen in Refs. [72], [73].

## 3 GAGA implementation

To optimize the performance of a thin planar nonreciprocal infrared absorber, we use a genetic algorithm with the intention of finding a global optimal convergence which meets the desired “fitness” without converging prematurely. GAs are known to be suitable for problems with objective functions that are not very computationally expensive. We calculate the absorptivity values, whose difference comprises the objective function, via an analytical recursion relation for the reflection coefficients (derivation shown in Appendix A). Owing to the analytical form of the solution, calculations take seconds per each design, making this approach compatible with the GA optimization process. We aim to maximize nonreciprocity at a given wavelength, quantified as a contrast in absorptance between two opposing angles of interest, such as −55° and 55° (Eq. (1)).

GA enables testing many different designs through a random initialization of parameters and can evolve the designs in response to a given figure of merit. This allows replacing less-efficient head-on approaches to design 1D magnetophotonic crystals such as trial-and-error or grid searches. GA is inspired by natural evolution and progresses through multiple stages of design – or so-called “child” creation steps – to ensure that the children are fitter than previous designs – the “parents.” The algorithm starts with a randomized population of bit-strings, which represent parameters of the absorber/emitter design. Similar to genetic evolution, the optimization process encompasses a representation, fitness evaluation, genetic mutation, crossover, all implemented for a specified number of iterations:

– **Representation** – the first step of GA implementation, which defines a pre-determined number of initial parent absorber structures in the population, e.g., schematically visualized in a bit-string format as parent1 = [11111], parent2 = [00000]…
− **Fitness evaluation** – identification of the best-performing structures by the FOM evaluation.
– **Selection** – the designs with the highest FOM values are selected for the next optimization step.
– **Crossover** – crossover is used to create children designs by stitching parts of two parent structures. E.g., from the two parents above, child1 = [11100] and child2 = [00011] can be generated, where the crossover point is random.
– **Genetic mutation** – the act of bit-flipping, which introduces random variability in the design, e.g., [00000] → [10000].

The number of members from the population that are selected, the crossover point in child generation, and mutation rates serve as probabilistic model hyperparameters to be tuned. The design parameters in our GA implementation take values that are stochastically generated within the pre-selected acceptable ranges. Used values of GA-specific parameters are: a population of 40, crossover rate of 0.9, mutation rate of 0.166, and if a mutation is specified, the probability of the WSM node separation vector direction flipping is 0.5.

### 3.1 Figure of merit

The main determining factor for the effectiveness of a GA to find an optimum design is the fitness evaluation/figure of merit (FOM) of the algorithm. In the Voigt configuration, the planar absorber response to s-polarized light is reciprocal (the electric field oscillations align with the magnetic field and their cross product becomes zero). Thus, we aim to minimize the absorptance of s-polarized light and maximize the contrast in the absorptance of p-polarized light incident at opposite angles. Achieving this goal calls for engineering absorber geometries that support p-polarized resonant modes in the frequency range where nonreciprocal response is desired and do not support any s-polarized modes in the same spectral range. These resonant modes (for both polarizations) can belong to the families of Fabry–Perot modes, photonic crystal cavity defect modes, Tamm interfacial states, or combinations thereof.

The absorptance contrast, Δ*α* (Eq. (1)), is commonly used as a FOM to measure the degree of nonreciprocity of absorber response to p-polarized radiation. Instead, we define the FOM as the ratio of the *α*_*H*_(*ω*_0_) to *α*_*L*_(*ω*_0_), where *H* and *L* are subscripts denoting the higher and lower absorptance values at opposite angles, respectively. This FOM includes both s- and p-polarized absorptance values explicitly (labeled by subscripts *s* and *p*):

(4) $$\begin{array}{ll} \text{FOM}=\frac{\alpha_{H}\left(\omega_{0}\right)}{\alpha_{L}\left(\omega_{0}\right)} & =\frac{\frac{\alpha_{S}\left(\omega_{0}\right)+\alpha_{P,H}\left(\omega_{0}\right)}{2}}{\frac{\alpha_{S}\left(\omega_{0}\right)+\alpha_{P,L}\left(\omega_{0}\right)}{2}}\\ & =\frac{\alpha_{S}\left(\omega_{0}\right)+\alpha_{P,H}\left(\omega_{0}\right)}{\alpha_{S}\left(\omega_{0}\right)+\alpha_{P,L}\left(\omega_{0}\right)}, \end{array}$$

where *ω*_0_ is the frequency corresponding to the maximum absorptance contrast for p-polarized waves:

(5) $$\omega_{0}={argmax}_{\omega }\left(\left|\alpha_{P,H}\left(\omega \right)-\alpha_{P,L}\left(\omega \right)\right|\right).$$

The inclusion of the s-polarized wave absorptance in the new FOM is critical, because any s-polarized contribution to thermal radiation would reduce the overall nonreciprocity of the structure by providing a parasitic reciprocal radiation channel. To maximize the nonreciprocity, we aim to minimize *α*_*S*_(*ω*_0_). The ideal nonreciprocal absorber/emitter would have *α*_*P*,*H*_(*ω*_0_) = 1 and *α*_*P*,*L*_(*ω*_0_) = 0, which maximizes Δ*α*. Under this scenario, FOM is reduced to $\frac{\alpha_{S}\left(\omega_{0}\right)+1}{\alpha_{S}\left(\omega_{0}\right)+0}$ , and this ratio is maximized when *α*_*S*_(*ω*_0_) approaches zero asymptotically.

It should be noted that in the non-Voigt configuration, an absorber can be engineered to resonantly enhance the s-to-p polarization conversion efficiency, thus enabling structures that exhibit nonreciprocal response for s-polarized light [38], [74].

Figure 4 visualizes the GA optimization process and provides insight as to how the FOM updates with the GA iterations. Each FOM update should increase or remain the same. Note that the design does not necessarily update with each iteration, since the next iteration is not always better than the previous. Figure 4 for instance was set to run for 20 iterations, but only updated 12 times.

**Figure 4:**
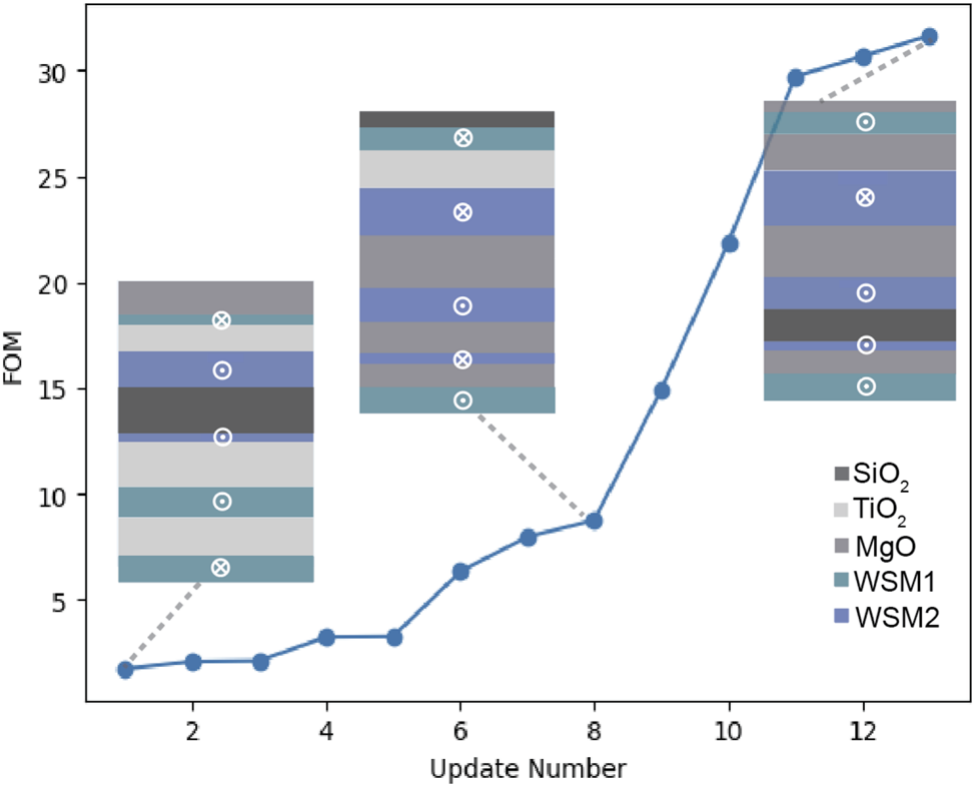
The graph shows 12 updates of the magnetophotonic crystal design produced by the GA, followed by examples of structures which produced 3 of the data points.

### 3.2 Gradient ascent method

In order to refine the final design, the structure generated by a global GA search was further optimized by using gradient ascent. This two-step genetic-algorithm-gradient-ascent (GAGA) optimization process retains the materials chosen by the genetic algorithm and aims to further optimize the layer thicknesses. Gradient ascent is an iterative first-order optimization algorithm, which aims to find local maxima of a differentiable function. Differently from the gradient descent method, gradient ascent algorithm maximizes the FOM instead of minimizing it. The algorithm evaluates the function’s gradient and updates the parameter values by moving in the direction of the steepest increase of the function. The learning rate hyperparameter determines how much parameter values are changed at each iteration and helps to balance between achieving convergence and reducing the computation time. The limitation of using the gradient ascent algorithm is that it guarantees finding a local maximum, but not necessarily a global one. Accordingly, the initial GA algorithm is necessary to explore a diverse design space in order to converge to a global maximum of FOM in the parameter space.

The equations for implementing gradient ascent to change the thicknesses for each layer are shown below [57]:

(6) $${\mathrm{grad}}_{i}=\frac{\Delta \alpha_{i}\left(d_{\text{old},i}+\Delta d\right)-\Delta \alpha_{i}\left(d_{\text{old},i}\right)}{\Delta d}$$

(7) $$d_{\text{new},i}=d_{\text{old},i}+d^{\prime }*\frac{{\mathrm{grad}\Delta \alpha }_{i}}{\left\|{\mathrm{grad}\Delta \alpha }_{i}\right\|}$$

In each equation, *i* is the layer number in the multilayer, Δ*α* is defined by Eq. (1), *d* is the thickness, and Δ*d* is the thickness variation. The difference in grad_*i*_ is calculated using an incremental increase Δ*d* of 2 nm per layer before being divided by this increment Δ*d*. The value *d*′ is the learning rate, which is 1.25 × 10^−9^ m in our implementation, and specifies the amount of change in the direction of the gradient.

A demonstration of the effect of this local optimization after 250 iterations is shown in Figure 5. The value of the absorptance contrast reaches an optimum value after about 150 iterations, before decreasing and subsequently plateauing. The initial absorptance start value of 0.901 has been improved to 0.953 using gradient ascent.

**Figure 5:**
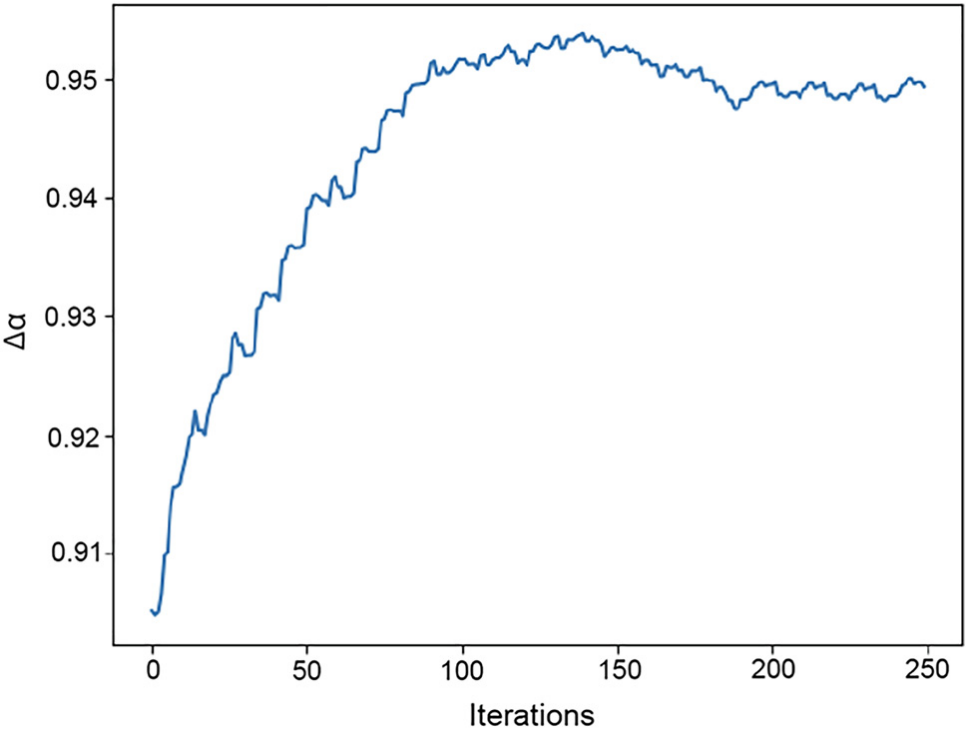
The numerical gradient ascent increases the absorptance at each layer with each iteration before finding a maximum and decreasing thereafter.

## 4 Final design

By using the parameters specified in previous sections, we were able to use the GAGA optimization to produce a final absorber design. The optimized absorber geometry is summarized in Table 2. Despite TiO_2_ being included as one of the five potential material candidates, the final design did not incorporate this material. One reason for this is the stochastic nature of the GA. Another reason could be that at the peak absorptance energy, TiO_2_ had the highest loss of the dielectrics (see Appendix B Figure 1). Furthermore, although the designs produced by the GA were set to include 10 layers, the final design has only 8. This is because the gradient ascent minimized the thicknesses of two layers to below 1 nm in value. As sub-nanometer layers are difficult to fabricate, we removed these layers from the final design. The FOM of the final GAGA-optimized 8-layer absorber structure differs negligibly from the initial GA-generated 10 layer structure yet requires fewer layers.

**Table 2:** The final design parameters. The table details the structure including the layer number, material (direction of the node separation for WSMs is indicated by ±2*b*), and thickness.

Layer #	Material	Thickness [nm]
1	WSM2 (+2*b*)	116
2	MgO	61
3	WSM1 (+2*b*)	215
4	SiO_2_	134
5	WSM1 (−2*b*)	309
6	WSM2 (+2*b*)	410
7	SiO_2_	103
8	WSM2 (−2*b*)	Semi-infinite

The GAGA algorithm can generate designs for any pre-determined number of layers, and there is no limit of the minimum number of layers. Our final design of 8 layers is a balance of practical fabricability and large nonreciprocity. We have found that decreasing the number of layers in the design leads to the decrease of the achieved nonreciprocity level, for example the maximum absorptance contrast for a 4 layer design is 0.6 (see Appendix C Table 3 for parameters of this design). Therefore, 8 layers is the minimum number of layers for our target high degree of nonreciprocity.

The absorptance spectra of the optimized absorber for incident angles of −55° (solid line) and 55° (dashed line) are plotted in Figure 6a. The maximum absorptance contrast of 0.953 has been achieved at 11.59 μm. Interestingly we notice that the optimum absorber spectra feature a second set of peaks, providing dual-channel nonreciprocity. Further examples of dual-channel nonreciprocity are seen in Refs. [22], [28]. Figure 6b shows a heatmap of the optimized structure absorptance as a function of frequency and angle of incidence, which spans both positive and negative values relative to the normal. This plot reveals that the structure supports multiple nonreciprocal modes formed as a result of the incident field interference with the waves reflected from different material interfaces. Comparing the photonic bandstructure in Figure 6b with the bandstructures of absorbers with composition identical to the final design but (i) with a nodal separation of zero (2*b* = 0, Appendix B Figure 3a) and (ii) with all the WSM node separation vectors pointing in the same direction (Appendix B Figure 3b), we observe coupling-induced mode splitting in Appendix B Figures 3a and b and 6b and magnetization-induced nonreciprocity in Appendix B Figures 3b and 6b, which is reinforced by the configurational symmetry breaking in our final design in Figure 6b. In Appendix B Figure 6, another design is shown, this time optimized at ±10° instead of ±55°; this demonstrates the effectiveness of our algorithm when optimized for small angles of incidence such as this.

**Figure 6:**
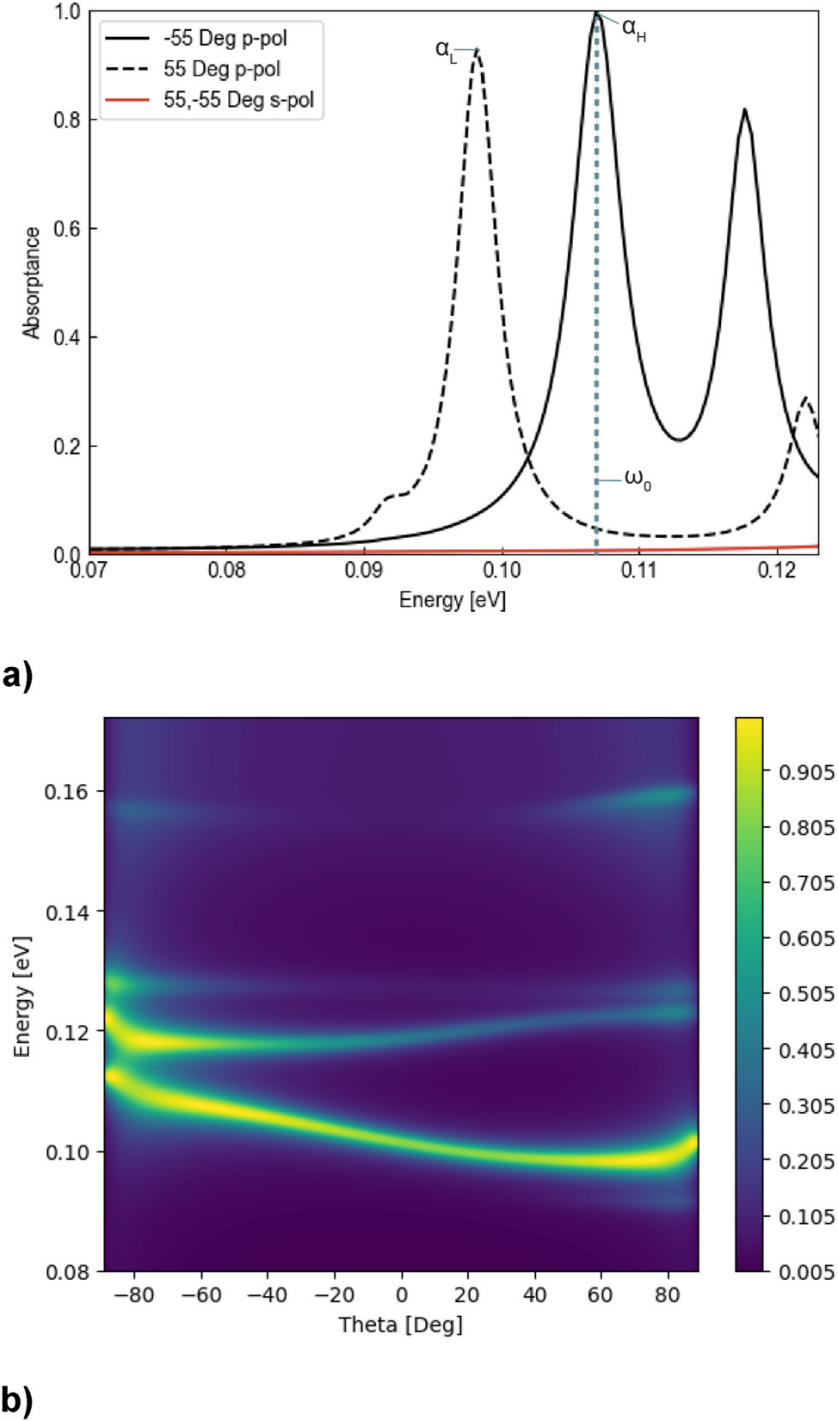
Absorptance spectra of the final structure for s- and p-polarizations and ±55° angles of incidence. Labels *α*_*L*_ and *α*_*H*_ indicate the low and high absorptance spectra used in the FOM definition. The largest absorptance contrast (and FOM) are observed at the frequency *ω*_0_ = 0.1069 eV. (a) Dispersion Heat map. (b) Heat map of the absorptance as a function of energy and angle of incidence.

The GAGA algorithm optimization takes a total of 3565.66 s to run (using the runtime parameters of the final design) on an Apple M2 Pro chip with ∼1 % of the overall CPU capacity and no GPUs, so this performance is expected on most laptops. The analytical absorptance calculation portion of the code takes 0.000276 s to run, which shows that our FOM is calculated very quickly to benchmark each design in the algorithm.

**Figure 7:**
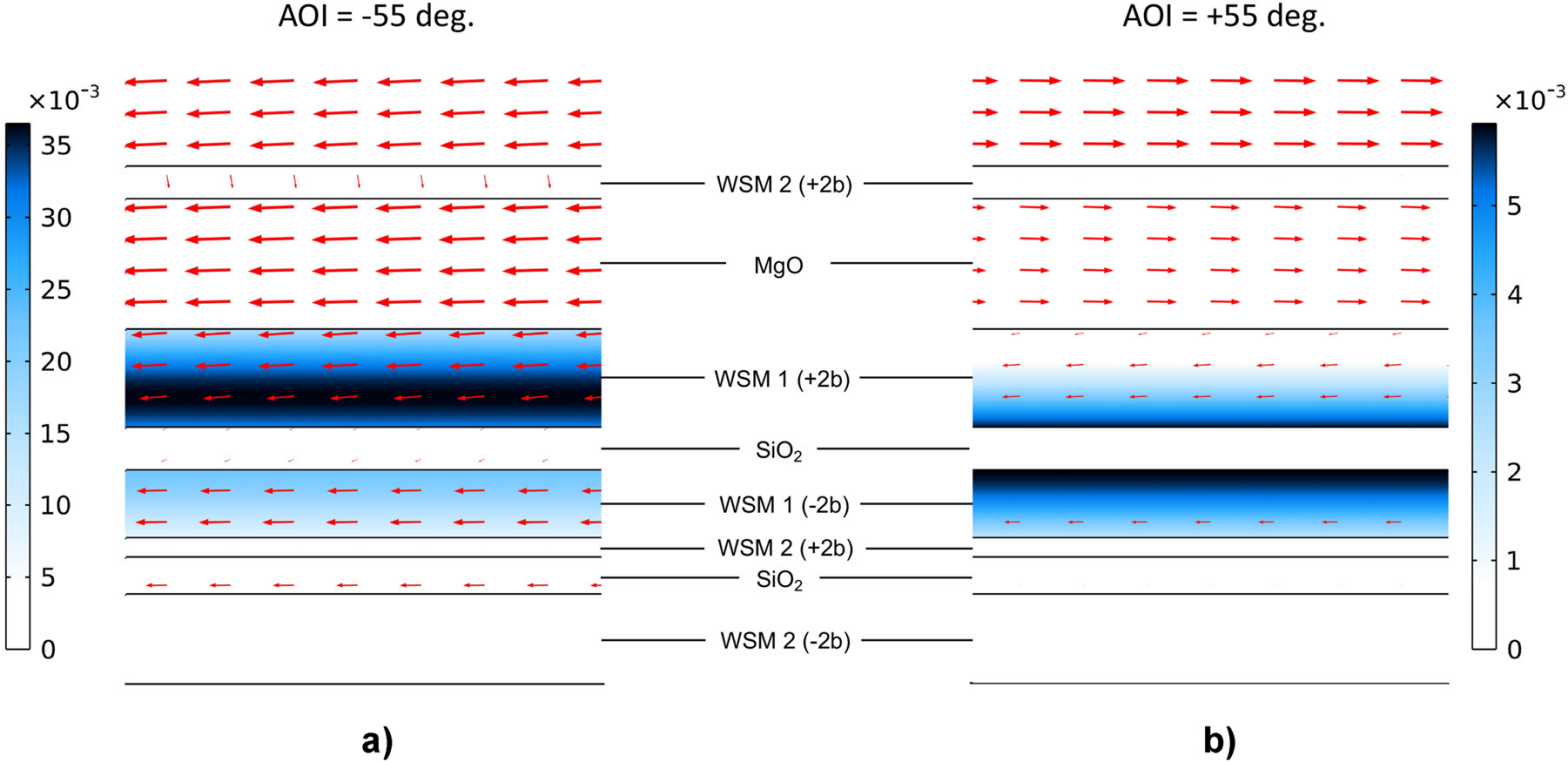
The electromagnetic energy absorbed per unit volume for (a) −55 and (b) +55° angles of incidence. The red arrows overlaying on the plot denote the magnitude and direction of the Poynting vector.

To reveal the physics of the light–matter interactions in the absorber that yield optimal nonreciprocity, we calculate the spatial energy profiles inside the structure using the finite element method (FEM) in COMSOL Multiphysics [75]. The spatial distributions of the electromagnetic energy absorbed per unit volume corresponding to *α*_*P*,*H*_(*ω*_0_) and *α*_*P*,*L*_(*ω*_0_) for both +55° and −55° angles of incidence are shown as heatmaps in Figure 7. For anisotropic media, the energy density is represented using the relation [76]:

(8) $$U=\frac{1}{2}\epsilon_{0}\bar{\bar{\epsilon }}\;E\cdot E^{*},$$

where *ϵ*_0_ is the free space permittivity, **E** is the electric field and $\bar{\bar{\epsilon }}$ is the permittivity tensor.

For lossy media, we can rewrite the permittivity tensor as $\bar{\bar{\epsilon }}={\bar{\bar{\epsilon }}}^{\prime }+i{\bar{\bar{\epsilon }}}^{\prime \prime }$ . Here, ${\bar{\bar{\epsilon }}}^{\prime }$ and ${\bar{\bar{\epsilon }}}^{\prime \prime }$ denote the real and imaginary parts of the permittivity tensor, respectively. The electromagnetic energy density dissipated at each spatial location can then be calculated as:

(9) $$W=\frac{1}{2}\epsilon_{0}{\bar{\bar{\epsilon }}}^{\prime \prime }E\cdot E^{*},$$

where *ϵ*_0_ is the free space permittivity, **E** is the electric field and ${\bar{\bar{\epsilon }}}^{\prime \prime }$ is the imaginary part of the permittivity tensor. The red arrows overlaying each of the distributions denote the magnitude and direction of the Poynting vector, revealing the flow of the electromagnetic energy in each case. Longer arrows indicate larger magnitudes, and all the arrows are shown on a logarithmic scale (Appendix B Figures 4 and 5).

Comparison of the plots in Figure 7a and b reveals that the contrasting structure response for the waves incident at −55° and +55° is driven by the configurational symmetry breaking in the part of the absorber comprised of layers 3–5, as predicted by the analysis in Figure 2. For −55° angle of incidence, most of the energy is dissipated in WSM1 layer 3, while for the opposite +55° angle, the energy dissipation mostly occurs in WSM1 layer 5, but is an order of magnitude smaller. This difference in the absorptance stems from selective coupling of incident waves to different-symmetry nonreciprocal modes supported by the structure. At lower frequencies, the structure reflects incident waves of both polarizations arriving at the surface at any angle (see, e.g., Additional Figure 4 for the electric field, absorptance and powerflow distributions in the structure at 0.08 eV).

**Figure 8:**
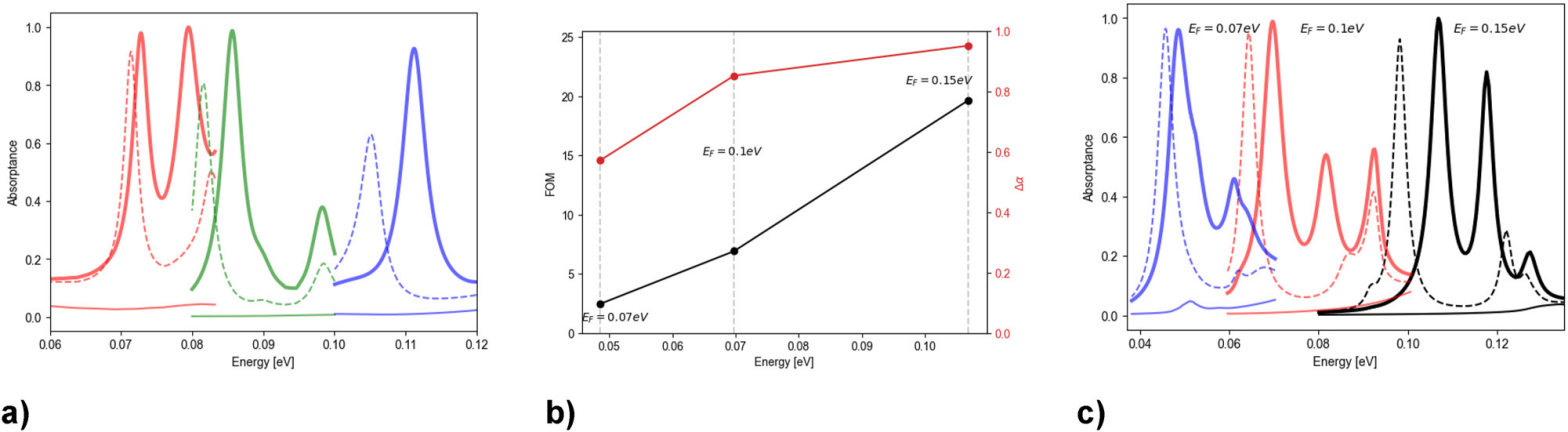
Tunability of the design. (a) GAGA-optimized spectra of the absorbers that exhibit nonreciprocity in the spectral ranges from 0.06 eV to 0.08 eV (red), 0.08 eV to 0.1 eV (green), and 0.1 eV to 0.12 eV (blue). Parameters for these designs can be found in Appendix C Table 1. (b) The FOM and absorptance contrast, Δ*α*, of the GAGA-optimized absorber with varied Fermi energy values of all the WSM1 and WSM2 layers (shown as labels). (c) The absorptance specta for the structures with different *E*_*F*_ values, where the dashed lines correspond to the −55° angle of incidence and p-polarization, the thick lines – 55° angle of incidence and p-polarization, and the thin lines, the ± 55° angle of incidence and s-polarization. The spectra with *E*_*F*_ = 0.15 eV (black curves) correspond of the final absorber design discussed in Figures 6 and 7. The graphs are truncated to include only their peaks for clarity of the figure.

To demonstrate the tunability of our design strategy, we have limited the allowed wavelength range to engineer multilayer absorbers with large nonreciprocal responses centered around a pre-defined photon energy value. In Figure 8a we plot the GAGA-generated spectra optimized for spectral ranges from 0.06 eV to 0.08 eV, 0.08 eV to 0.1 eV, and 0.1 eV to 0.12 eV. Each of these plots exhibits high nonreciprocity in a pre-defined spectral region. The frequency spectrum of the nonreciprocal absorber can also be dynamically tuned by changing the Fermi energy of WSM materials included in the design. Reference [77] found that *E*_*F*_ was one of the more influential parameters when it came to changing the WSM permittivity function. Topological semimetals such as the WSMs used in this work have been known to have tunable Fermi energy through the use of nanostructures and gate voltages [78], [79], [80]. They can also be tuned thermally because of the Fermi energy’s temperature dependence [81] caused by the increase or decrease of the charge carrier density [82]. The plot in Figure 8b and c shows how the absorption spectra of the optimized emitter change with increasing Fermi energy of both WSMs. The chosen values are *E*_*F*_ = 0.07 eV, 0.1 eV, and 0.15 eV, inspired by parameters described in earlier works [5], [46], [83]. As a result, different operating wavelengths can be accessed by tuning the Fermi energy. Since we are limited by the available data for MgO (which do not exist beyond 0.1238 eV [41]), we are unable to use *E*_*F*_ values much higher than those seen in Figure 8b and c. In calculating the spectral shifts in Figure 8b and c, we make the assumption used in prior works that the Fermi energy, *E*_*F*_, can be tuned independently of other WSM parameters [7], [77].

## 5 Conclusions

To summarize, we used a genetic algorithm followed by the gradient ascent fine-tuning procedure, together known as GAGA, to design an ultra-thin planar nonreciprocal absorber with the absorptance contrast competitive with prior designs, but achieved in a much more compact planar format (see Figure 1). Our final design has sub-wavelength thickness and consists of only 8 layers, while offering a high absorptance contrast of 0.953. The absorber includes thin-film WSMs as well as dielectrics SiO_2_ and MgO, and achieves efficient performance by breaking configurational symmetry of the structure, which results in selective coupling of the p-polarized waves incident at opposite angles to different-symmetry modes in the structure. The choice of a new FOM that takes both s- and p-polarization contributions into account allows to optimize the overall thermal absorber/emitter efficiency. By using the new FOM, we generated designs with high absorptance contrast in the p-polarizations accompanied by the negligible s-polarization absorptance. The final design showed a dual channel with both wide-angle and narrow-band nonreciprocity. We used FEM simulations to illuminate the physics of the structure and found that the variation in absorptance is owed to the selective coupling of incident light with non-reciprocal modes that have differing symmetries. Our optimized planar absorber/emitter is magnet-less, thin, lithography-free, and dynamically-tunable, paving the way for demonstration of compact, high-performance WSM-based thermal emitters [5], [6], [7], antennas [10]–[14], optical isolators [44], and switches [43] in the infrared regime.

## Figures and Tables

**Figure 1:**
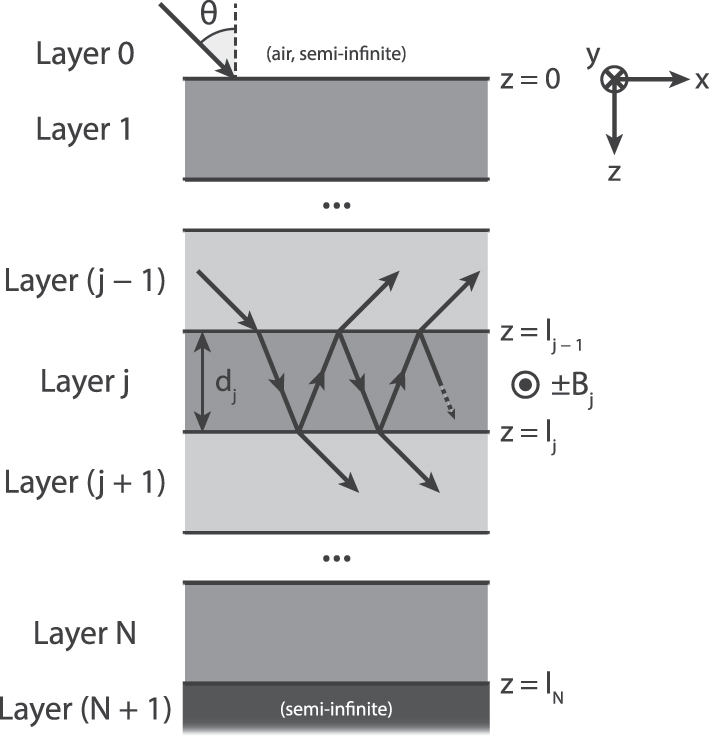
Schematic of an *N*-layer 1D magnetophotonic crystal in the Voigt configuration, with the 0th layer being air and the (*N* + 1)th layer being the substrate. Light is incident from layer 0 at an angle of incidence *θ*. Multiple reflections in layer *j* as a result of an incident wave from layer (*j* − 1) are shown. The external magnetic field in the *j*th layer, *B*_*j*_ can point into or out of the plane of incidence.

**Figure 1:**
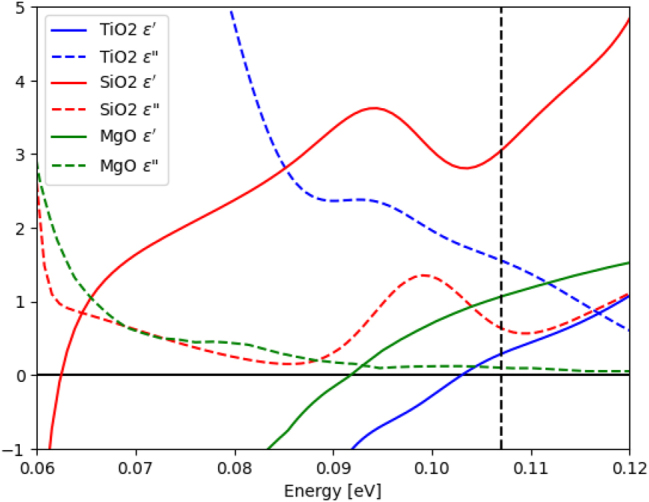
Dielectric functions of SiO_2_, TiO_2_, and MgO, where *ϵ*′ and *ϵ*″ are the real and the imaginary parts of the dielectric function, respectively. The dashed black line marks the frequency of the maximum absorptance contrast, Δ*α*.

**Figure 2:**
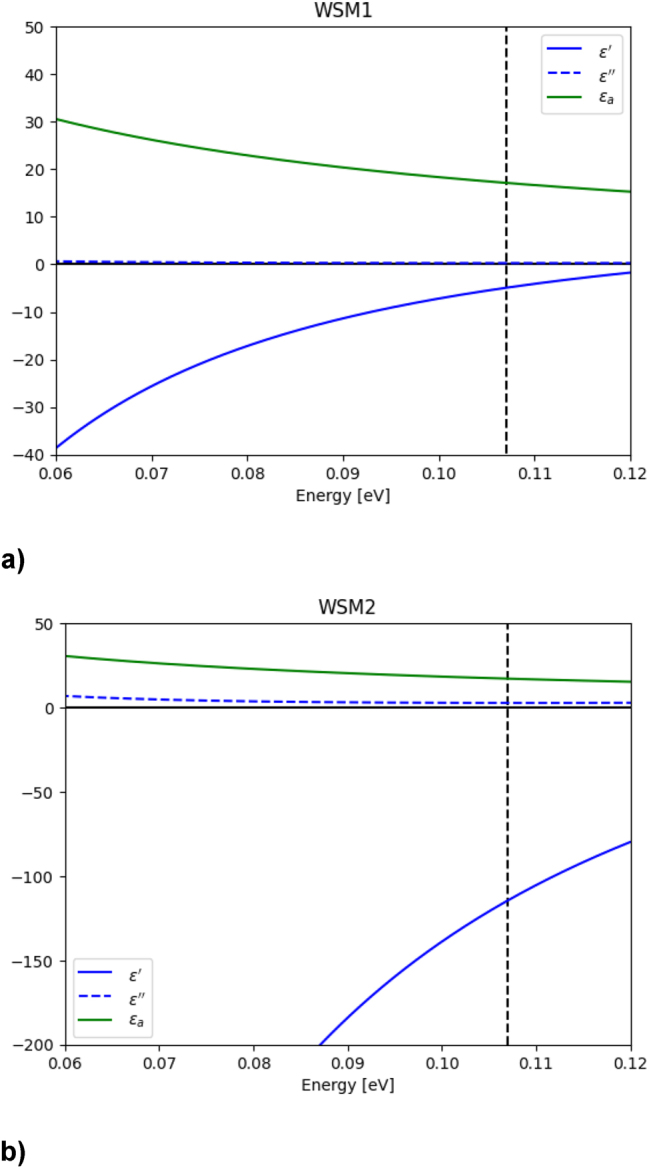
Weyl semimetal dielectric function plots. (a) and (b) Are the dielectric functions of WSM1 and WSM2 where *ϵ*′ and *ϵ*″ are the real and the imaginary parts of the diagonal components of the dielectric tensor the WSM1/WSM2 materials (*ϵ*_*ii*_ = *ϵ*′ + *ϵ*″), and *ϵ*_*a*_ is the off-diagonal contribution. The dashed black line marks the frequency of the maximum absorptance contrast, Δ*α*.

**Figure 3:**
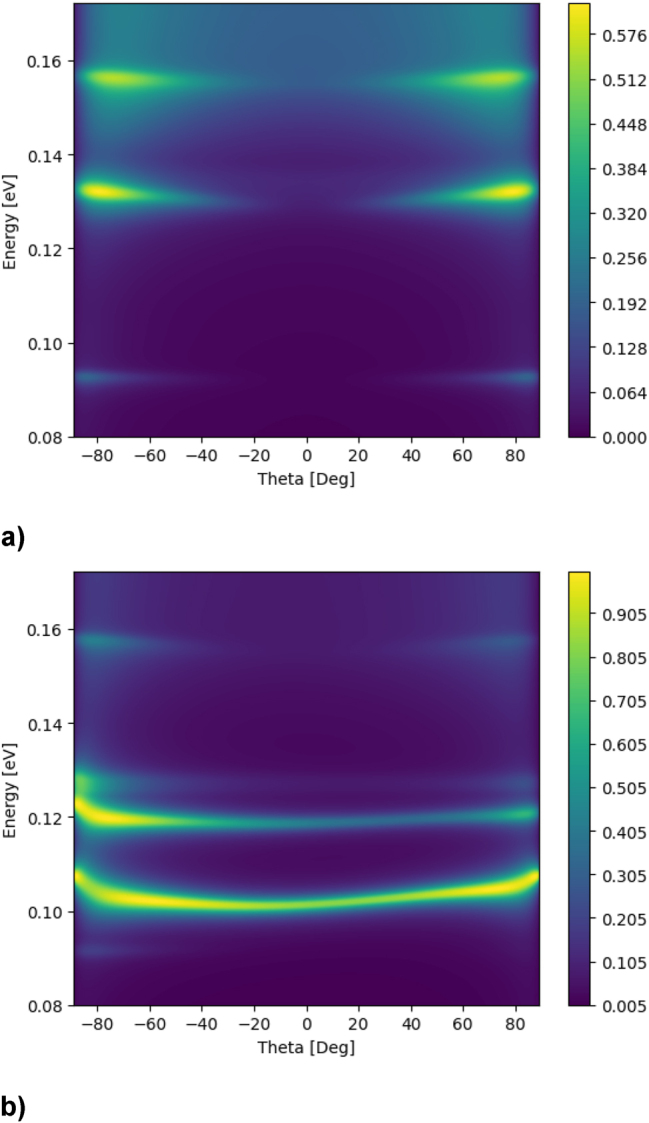
Heatmaps of the absorptance of the final structure under varying magnitude and directions of the 2 $\vec{b}$ vectors in the WSM layers. (a) The absorptance spectrum for the structure identical to the final design but with *b* = 0 for both WSMs, making the design reciprocal. (b) Same is in (a), but for the case of the vectors 2 $\vec{b}$ having the same orientation (+2*b*). Increased configurational symmetry makes this absorber response more reciprocal than that of our final design shown in Figure 5b.

**Figure 4:**
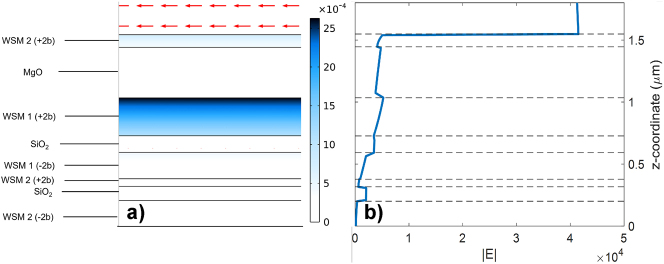
The electromagnetic energy absorbed per unit volume at 0.08 eV with p-polarized incident light, where non-reciprocity is not prominent. The red arrows overlaying on the plot denote the magnitude and direction of the Poynting vector.

**Figure 5:**
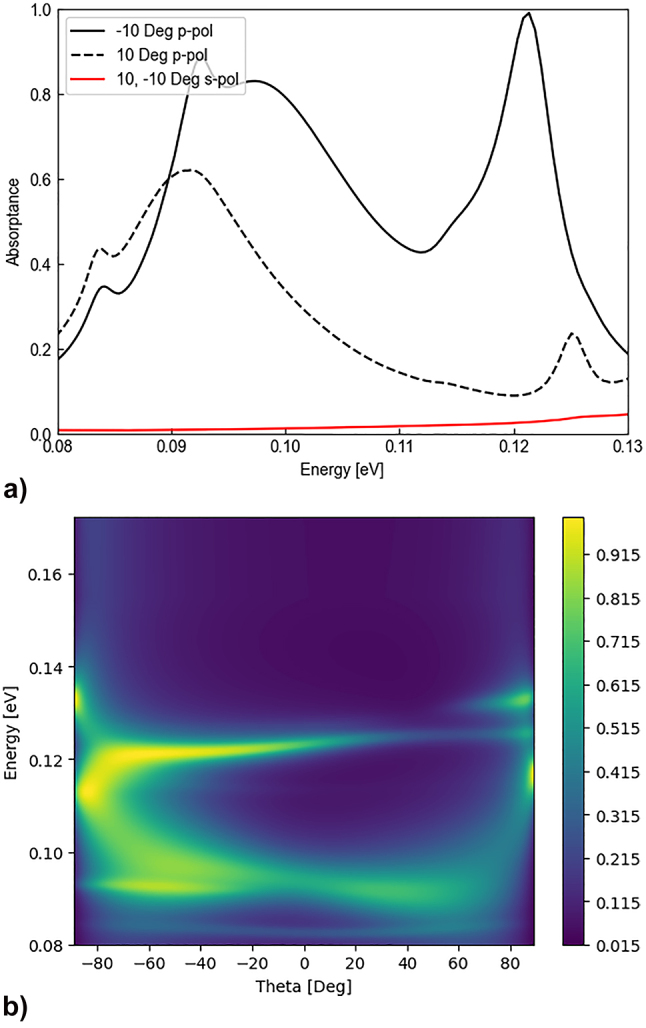
Absorptance spectra of the final structure for s- and p-polarizations and ±10° angles of incidence. Labels *α*_*L*_ and *α*_*H*_ indicate the low and high absorptance spectra used in the FOM definition. (b) Heat map of the absorptance as a function of energy and angle of incidence. See Appendix C for parameter specifications.

**Figure 6:**
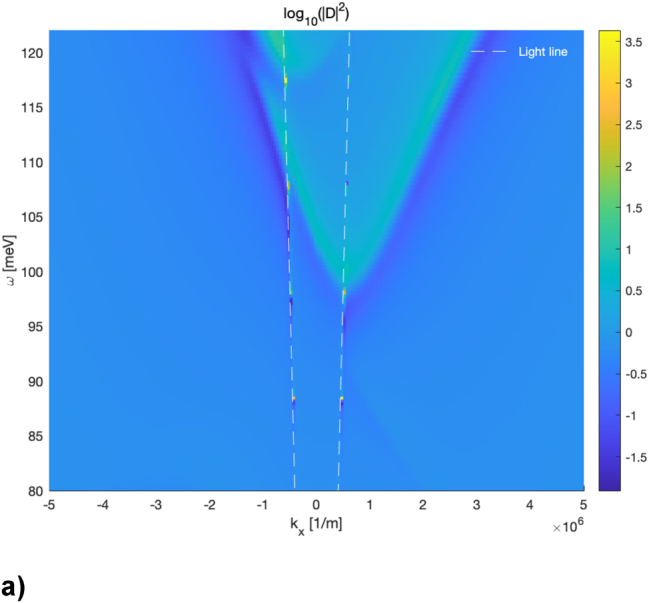
Heat map of the log(|*D*|^2^) (with omega on the *y*-axis and *k*_*x*_ on the *x*-axis) for the final design where *D* is the denominator of the reflection and transmission coefficients like in Equations (11) and (13). This plot is able to show the dispersion relation of the final design numerically.

## Appendix A: Reflection and transmission coefficients of a 1D magnetophotonic crystal

In this work, the reflection and transmission coefficients of the multilayer structures (i.e., 1D magnetophotonic crystals in the Voigt configuration) were calculated using a recursion relation. This is described, for example, in Ref. [84]. This approach works well because the magnetooptical layers (i.e., the Weyl semimetals) happen to support s- and p-polarized waves in the Voigt configuration, which facilitates the calculation of the single-interface reflection and transmission coefficients because there is no polarization conversion (*r*_*s*,*p*_ = *r*_*p*,*s*_ = 0). Note that one complication unique to this approach is that the single-interface reflection and transmission coefficients are nonreciprocal, i.e., at the interface between layers *a* and *b*, *r*_*a*,*b*_(+*k*_*x*_) ≠ *r*_*a*,*b*_(−*k*_*x*_) and *t*_*a*,*b*_(+*k*_*x*_) ≠ *t*_*a*,*b*_(−*k*_*x*_); likewise, *r*_*b*,*a*_(+*k*_*x*_) ≠ *r*_*b*,*a*_(−*k*_*x*_) and *t*_*b*,*a*_(+*k*_*x*_) ≠ *t*_*b*,*a*_(−*k*_*x*_) (here, *k*_*x*_ is the *x*-component of the wavevector, parallel to the interfaces).

We verified that the recursion relation, to be shown, is equivalent to the transfer matrix method [85]–[88] commonly used in the literature. In what follows, we describe the key equations used to calculate the reflectance *R* and transmittance *T* of the designs in this work and hence the absorptance *A* = 1 − *R* − *T* (from conservation of energy).

Consider the *j*th layer in a 1D magnetophotonic crystal, as shown in Figure 1. The total reflection coefficient of the (*j* − 1)th layer is defined as the ratio of the *x*-components of the reflected and incident electric fields, accounting for multiple reflections in all the ≥j layers below: Γ_*j*−1_ = *E*_*xr*_/*E*_*xi*_. *E*_*xr*_ can be written in terms of *E*_*xi*_ and the single-interface reflection and transmission coefficients, accounting for nonreciprocity and accumulating phase with each round trip:

(10)
$$\begin{array}{ll} E_{xr} & =r_{j-1,j}E_{xi}+t_{j-1,j}r_{j,j+1}t_{j,j-1}e^{2ik_{zj}d_{j}}E_{xi}\\ & \;+t_{j-1,j}r_{j,j+1}r_{j,j-1}r_{j,j+1}t_{j,j-1}e^{4ik_{zj}d_{j}}E_{xi}+\cdots \end{array}$$

Here, *r*_*a*,*b*_ and *t*_*a*,*b*_ are the single-interface reflection and transmission coefficients between layers *a* and *b*, respectively, *k*_*zj*_ is the *z*-component of the wavevector in layer *j*, and *d*_*j*_ is the thickness of layer *j*. Eq. (10) is an infinite geometric sum, allowing us to simplify it to the following:

(11)
$$\Gamma_{j-1}=r_{j-1,j}+t_{j-1,j}t_{j,j-1}\frac{\Gamma_{j}e^{2ik_{zj}d_{j}}}{1-r_{j,j-1}\Gamma_{j}e^{2ik_{zj}d_{j}}},$$

where we have replaced *r*_*j*,*j*+1_ by Γ_*j*_. Equation (11) is a recursion relation that relates the total reflection coefficient of any given layer to that of the layer under it. If the system consists of *N* layers, where the semi-infinite 0th layer contains the incident and reflected waves and the semi-infinite (*N* + 1)th layer contains the transmitted wave, the recursion relation is initialized by Γ_*N*_ = *r*_*N*,*N*+1_ (or equivalently, Γ_*N*+1_ = 0. If the 0th layer is isotropic and lossless (e.g., air or vacuum), the reflectance of the multilayers structure is given by *R* = |Γ_0_|^2^.

For the transmittance, the approach is similar. Define a transmission coefficient from the (*j* − 1)th layer to the *j*th layer, *S*_*j*−1,*j*_, as the ratio of the *x*-component of the transmitted and incident electric fields at *z* = *ℓ*_*j*−1_. Once again, this can be written in the form of a geometric sum:

(12)
$$\begin{array}{ll} E_{x}^{\left(j\right)}e^{ik_{zj}\ell_{j-1}} & =t_{j-1,j}E_{x}^{\left(j-1\right)}e^{ik_{zj-1}\ell_{j-1}}+t_{j-1,j}r_{j,j+1}\\ & \;\times r_{j,j-1}e^{2ik_{zj}d_{j}}E_{x}^{\left(j-1\right)}e^{ik_{zj-1}\ell_{j-1}}\\ & \;+t_{j-1,j}r_{j,j+1}r_{j,j-1}r_{j,j+1}r_{j,j-1}e^{4ik_{zj}d_{j}}\\ & \;\times E_{x}^{\left(j-1\right)}e^{ik_{zj-1}\ell_{j-1}}+\cdots \end{array}$$

This infinite geometric sum simplifies to

(13)
$$S_{j-1,j}=\frac{t_{j-1,j}}{1-r_{j,j-1}\Gamma_{j}e^{2ik_{zj}d_{j}}},$$

where we have once again replaced *r*_*j*,*j*+1_ by Γ_*j*_. Having Eq. (13), we can determine the total transmission coefficient of the *N*-layer system. Consider the following: when the incident electric field $E_{x}^{\left(0\right)}$ propagating in the 0th layer reaches the interface at *z* = *ℓ*_0_, it is multiplied by *S*_0,1_. It then propagates in the 1st layer, accumulating phase $e^{ik_{z1}\left(\ell_{1}-\ell_{0}\right)}$ until it reaches the interface at *z* = *ℓ*_1_ and is multiplied by *S*_1,2_. The process repeats itself, and the transmitted electric field propagating in the (*N* + 1)th layer is

(14)
$$E_{x}^{\left(N+1\right)}e^{ik_{zN+1}\ell_{N}}=E_{x}^{\left(0\right)}e^{ik_{z0}\ell_{0}}\left(\overset{N}{\underset{j=0}{\prod }}\theta_{j-1,j}\right)S_{N,N+1},$$

where $\theta_{j-1,j}=S_{j-1,j}e^{ik_{zj}d_{j}}$ . Then, by inspection, the total transmission coefficient is

(15)
$$\Theta =\left(\overset{N}{\underset{j=1}{\prod }}\theta_{j-1,j}\right)t_{N,N+1}.$$

The total transmittance can be derived using Poynting flux arguments. If the time-averaged Poynting flux in the *z*-direction is given by $S_{z}=\frac{1}{2}R\left[\hat{z}\cdot \left(\vec{E}\times {\vec{H}}^{*}\right)\right]$ , it can be shown that

(16)
$$\begin{array}{cc} S_{zi}^{\left(0\right)} & =\frac{1}{2}\frac{\epsilon^{\left(0\right)}k_{0}}{Zk_{z0}}|E_{xi}^{\left(0\right)}{|}^{2},\\ S_{zt}^{\left(N+1\right)} & =\frac{1}{2}R\left[\frac{\epsilon^{\left(N+1\right)}}{k_{zN+1}}\right]\frac{k_{0}}{Z}|E_{xt}^{\left(N+1\right)}{|}^{2}. \end{array}$$

*Z* is the impedance of free space. The transmittance is defined as $T=S_{zt}^{\left(N+1\right)}/S_{zi}^{\left(0\right)}$ . Thus,

(17)
$$T=\frac{R\left[\epsilon^{\left(N+1\right)}/k_{zN+1}\right]}{\epsilon^{\left(0\right)}/k_{z0}}|\Theta {|}^{2}.$$

If the multilayer structure is suspended in air (i.e., the semi-infinite 0th and (*N* + 1)th layers are in air), *T* = |Θ|^2^.

In all of the above expressions, the most general way to write the single-interface reflection and transmission coefficients (i.e., between two magnetooptical materials in the Voigt configuration) is:

(18)
$$\begin{array}{c} r=\frac{\epsilon_{1}^{NR}\left(+2b\right)-\epsilon_{2}^{NR}\left(+2b\right)}{\epsilon_{1}^{NR}\left(-2b\right)+\epsilon_{2}^{NR}\left(+2b\right)},\\ t=\frac{2\epsilon_{1}^{t}\left(k_{z2}^{0}/k_{z1}^{0}\right)k_{z1}}{\epsilon_{1}^{NR}\left(-2b\right)+\epsilon_{2}^{NR}\left(+2b\right)}, \end{array}$$

where $\epsilon_{i}^{NR}\left(\pm 2b\right)=\epsilon_{i}^{t}\frac{k_{zi}}{k_{zi}^{0}}\mp ig_{i}\frac{k_{x}}{k_{z1}^{0}}$ and $k_{zi}^{0}=k_{zi}\left(g_{i}\to 0\right)$ . Equation (18) is derived similarly to the standard reflection and transmission coefficients (i.e., for isotropic materials), but accounting for the fact that the polarization ratio (*E*_*z*_/*E*_*x*_) depends on the off-diagonal components of the dielectric tensor. The same expressions for *r* and *t* cast in a different form can be found in Ref. [85].

## Appendix C: Additional tables

**Table 1:** Design parameters for Figure 8a designs. The table details the structure including the layer number, material (direction of the node separation for WSMs is indicated by ±2*b*), and thickness. GAGA generated designs with near-zero thickness were taken out.

Figure 8a 0.06 eV–0.08 eV		
Layer #	Material	Thickness [nm]
1	MgO	956
2	WSM1 (+2*b*)	865
3	SiO_2_	215
4	WSM1 (−2*b*)	186
5	MgO	1026
6	WSM1 (+2*b*)	764
7	MgO	657
8	WSM2 (+2*b*)	323
9	TiO_2_	127
10	WSM2 (+2*b*)	Semi-infinite

Figure 8a 0.08 eV–0.01 eV		
Layer #	Material	Thickness [nm]
1	SiO_2_	61
2	WSM2 (−2*b*)	52
3	WSM2 (+2*b*)	42
4	SiO_2_	41
5	WSM1 (+2*b*)	717
6	MgO	1047
7	WSM1 (−2*b*)	95
8	MgO	817
9	WSM2 (−2*b*)	Semi-infinite

Figure 8a 0.01 eV–0.012 eV		
Layer #	Material	Thickness [nm]
1	SiO_2_	197
2	WSM2 (+2*b*)	192
3	WSM1 (+2*b*)	257
4	SiO_2_	162
5	WSM1 (−2*b*)	35
6	SiO_2_	393
7	WSM2 (+2*b*)	1029
8	SiO_2_	952
9	WSM2 (+2*b*)	Semi-infinite

**Table 2:** Design parameters for Appendix B Figure 6 design. The table details the structure including the layer number, material (direction of the node separation for WSMs is indicated by ±2*b*), and thickness. Thicknesses with near-zero thickness were taken out.

Appendix B Figure 6 optimized for ±10°		
Layer #	Material	Thickness [nm]
1	WSM1 (+2*b*)	67
2	MgO	99
3	WSM1 (−2*b*)	271
4	TiO_2_	151
5	WSM2 (+2*b*)	178
6	TiO_2_	634
7	WSM1 (+2*b*)	681
8	SiO_2_	819
9	WSM2 (−2*b*)	Semi-infinite

**Table 3:** Design parameters for 4 layer design. The table details the structure including the layer number, material (direction of the node separation for WSMs is indicated by ±2*b*), and thickness. Thicknesses with near-zero thickness were taken out.

4 layer design		
Layer #	Material	Thickness [nm]
1	MgO	480
2	WSM1 (+2*b*)	273
3	SiO_2_	222
4	WSM2 (+2*b*)	Semi-infinite
